# Recruitment strategies for reaching adults aged 50 years and older with low socioeconomic status for participation in online physical activity interventions

**DOI:** 10.3389/fdgth.2024.1335713

**Published:** 2024-05-10

**Authors:** Eline H. G. M. Collombon, Catherine A. W. Bolman, Gert-Jan de Bruijn, Denise A. Peels, Lilian Lechner

**Affiliations:** ^1^ Faculty of Psychology, Open Universiteit, Heerlen, Netherlands; ^2^ Department of Communication Studies, University of Antwerp, Antwerp, Belgium

**Keywords:** older adults, low education, vulnerable populations, eHealth, mHealth

## Abstract

**Background:**

Generally, the health condition of those with higher socioeconomic status (SES) is better compared to those with lower SES. The application of appropriate strategies to reach low SES populations with electronic health (eHealth) interventions is thus of major importance to reduce health inequalities. eHealth-studies providing detailed information on recruitment strategies are scarce, despite the fact that this information is crucial for comparable research and implementation.

**Objective:**

To provide insight into the reach, sample characteristics and costs of three pre-planned strategies for recruiting adults aged 50 years and older with low SES for participation in an online physical activity intervention, as part of a field study.

**Methods:**

Recruitment took place via (1) invitation letters via a municipality, (2) gyms and (3) social media advertisements, aiming to include 400 participants. Additional procedures were followed to reach specifically the low SES group. Response rates, sociodemographic characteristics and costs per strategy were assessed.

**Results:**

The highest response was shown for the municipality approach (*N* = 281), followed by social media (*N* = 71) and gyms (*N* = 45). Ten participants were recruited via family/friends. The most low-educated participants were reached via the municipality (*N* = 128) followed by social media (*N* = 9), gyms (*N* = 8) and family/friends (*N* = 5). Recruitment costs were with €2,142.37 the highest for the municipality compared to €96.81 for social media and no costs for gyms.

**Conclusions:**

Recruitment via invitation letters through a municipality has the highest potential for reaching low SES participants of the three applied strategies, although the higher recruitment costs need to be taken into account.

## 1 Introduction

Socioeconomic status (SES) is a term used to describe an individual's affluence or social standing, referencing factors such as wealth, educational level and occupation (1). The relationship between SES and health has been well-established, and shows that the health condition of those with higher SES is better compared to those with lower SES (2, 3). The influence of SES on health is closely related to people's lifestyle which includes health risk behaviors such as smoking and health promoting behaviors such as physical activity (PA) (4). Given this, it is remarkable that lifestyle-related electronic health (eHealth) and mobile health (mHealth) interventions are often not used by the low SES population (5). Although challenging, reaching participants with low SES with electronic health (eHealth) interventions is, particularly in the light of the current digital era, of major importance to reduce health inequalities due to SES instead of widening the gap.

An important step for reaching participants with low SES is the application of well-planned, appropriate and inclusive recruitment strategies. eHealth-studies providing detailed information on strategies applied to reach their participants are scarce, despite the fact that this information is crucial for comparable research. Available literature on recruitment strategies often focuses on clinical settings, randomized controlled trials (RCTs), not specifically on eHealth research and/or the SES group (6–9). Therefore, the aim of the current study was to provide insight into the reach, sample characteristics and costs of three pre-planned strategies for recruiting adults aged 50 years and older with low SES for participation in an online PA intervention consisting of three computer-based tailored PA advices combined with a mobile-based activity tracker (10), as part of a field study. To accomplish this, the following research questions (RQ) were investigated: (RQ1) Which recruitment strategy results in the highest and fastest response?, (RQ2) Which recruitment strategy is most suitable for reaching the low SES population?, (RQ3) Which recruitment strategy is most suitable for reaching populations with a specific gender, age or health status?, (RQ4) Which recruitment strategy is most beneficial with regard to costs?.

## 2 Methods

### 2.1 Participants

The aim for the field study was to include 400 participants aged 50 years and older with a focus on the low SES population, although other SES groups were not excluded from participation. An additional aim was that the sample consisted of 200 participants without a (chronic) disease and 200 participants with a (chronic) disease, since tailoring procedures within the online PA intervention were optimized prior to the field study specifically for these subgroups. With this study population distribution, detailed insights on use and appreciation of the intervention for subgroups with and without (chronic) diseases could be obtained, which is described elsewhere [in preparation] A criterium for being classified into the (chronic) disease group was that participants were limited in being physically active as a result of their disease. Additional inclusion criteria applicable to the total sample were: (1) able to use a computer, laptop or tablet, (2) having an e-mail address, (3) having a smartphone, (4) not previously participated in a study of the Active4Life project (11). Eligibility to participate was assessed during the online registration procedure. The aim of this paper was to provide insight into the recruitment strategies applied during the field study with a specific focus on reaching low SES groups.

### 2.2 Recruitment procedures

Three different pre-planned recruitment strategies were deployed parallel to each other, namely recruitment via (1) a municipality, (2) gyms and (3) social media. These strategies were selected and considered appropriate and feasible based on previous eHealth studies conducted within our research group (12, 13).

Firstly, residents aged 50 years and older of three low SES neighbourhoods in a municipality received a personal invitation letter by post on behalf of the municipality, VIE (a regional organization that stimulates lifestyle and vitality) and the university for participation in the online PA intervention. The letters were basically the same for all invited residents, but differed in detail for the age groups 50–64 years and 65 + years. The 50–64 letter focused more on the healthy population, whereas the 65 + letter focused more on the population with health complaints. However, in both letters it was emphasized that anyone aged 50 years and older could participate despite any health problems. Interested invitees could register via internet by entering the hyperlink stated in the paper-based invitation letter.

Secondly, recruitment took place via gyms affiliated with project partner NL Actief, the Dutch trade association for sports organizations. Gyms were invited by NL Actief to participate in the recruitment procedures based on their location in a low SES region. Gyms signing up to participate, received an online flyer to distribute among their (potential) members aged 50 years and older. Methods for distribution of the flyer were determined by gyms themselves, although options were provided by the researchers for guidance. Some gyms included the flyer for example in their newsletter, whereas others posted the flyer on their social media channels. Since the flyers were online, interested people could be directly forwarded to the information website and registration portal by clicking on the link in the flyer.

The third strategy was recruitment via social media advertisements on Facebook. In order to reach participants aged 50 years and older with low SES, three targeting settings on age, educational level and location were added to the advertisements. With regard to age, the advertisement was only shown to adults aged 50 years and older. For educational level, the advertisement was not shown to those who added to their Facebook profile that they were attending higher education or a master degree or those who received their higher education certificate, university degree, master degree or PhD. The advertisements were only shown within pre-selected Dutch regions based on the number of low-educated persons living in the area combined with the degree of ageing (14). Also here, interested people were directly forwarded to the information website and registration portal by clicking on the link in the online flyer.

### 2.3 Measures and statistics

Recruitment method and the sociodemographics gender, age, educational level and (chronic) disease were assessed during the registration procedure. Educational level was categorized into low (i.e., primary, basic vocational or lower general school), middle (i.e., medium vocational school, higher general secondary education and preparatory academic education) and high (i.e., higher vocational school or university level) according to the Dutch educational system (15). Participants were classified into the (chronic) disease subgroup when they indicated during the registration procedure that they were limited in being physically active as a result of a (chronic) disease. Performance statistics of social media advertisements such as costs, reach and clicks on links were derived from the ad center of Facebook. Information on costs and reach of the personal invitation letters was provided by the municipality. Chi-squares and one-way analyses of variances (ANOVAs) were performed to test on an exploratory level for differences on the above mentioned sociodemographics between the recruitment strategies (*P* ≤ .05).

## 3 Results

### 3.1 Which recruitment strategy results in the highest and fastest response?

**Figure 1:**
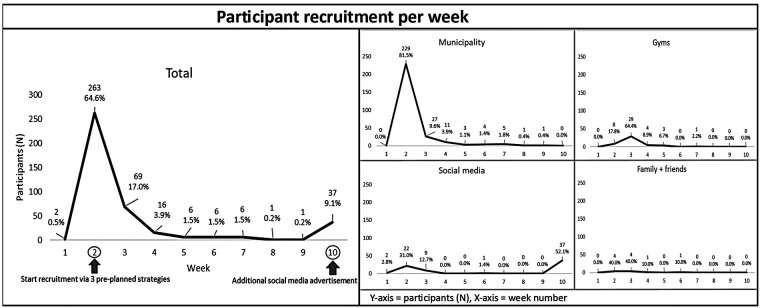
Overview of number of recruited participants per week.

Recruitment via the three pre-planned strategies was initiated in week 2. The participants that were recruited in week 1 (*N* = 2) were reached via social media, likely via advertisements for previous already completed studies of the Active4Life project where the same registration portal was used (11). The most participants were also recruited in the second week when pre-planned strategies were initiated (*N* = 263). Of those 263 participants, the majority was reached via the municipality (*N* = 229). In addition to the three pre-planned recruitment strategies, 10 participants indicated that they came into contact with the online PA intervention by family, friends or acquaintances. This unplanned and naturally developed recruitment method is reported as the fourth strategy. A schematic overview of the number of recruited participants per week in total and separated per strategy is provided in Figure 1. In the third week, 69 participants were recruited with the majority coming from gyms (*N* = 29). Afterwards, the number of recruited participants per week decreased gradually. With only 1 recruited participant in both the eighth and ninth week, it was decided that a new action was needed to boost recruitment. Since only 30 additional participants were needed to reach the planned sample size of 400, solely recruitment via social media was boosted through a new advertisement. No new actions were performed within the municipality and gyms approach, to avoid an overload of recruited participants. As a result, 37 participants were recruited via social media in the tenth week resulting in a total of 407 registered adults aged 50 years or older. No selection of recruited participants was needed to reach the pre-defined goal of including 200 participants with a (chronic) disease and 200 participants without a (chronic) disease. This distribution within the study population arose naturally during recruitment. The municipality approach delivered the most participants (*N* = 281, 69.0% of the total study population), followed by social media (*N* = 71, 17.4%), gyms (*N* = 45, 11.1%) and family + friends (*N* = 10, 2.5%).

### 3.2 Which recruitment strategy is most suitable for reaching the low SES population?

Sociodemographic characteristics of recruited participants in total and per strategy are shown in Table 1. In total, more low- (*N* = 150, 36.9% of the total study population) and middle-educated participants (*N* = 149, 36.6%) were reached than high-educated participants (*N* = 108, 26.5%). Significantly more low-educated participants were reached via the municipality (*N* = 128, 45.6% of the municipality group) than via gyms (*N* = 8, 17.8%) and social media (*N* = 9, 12.7%) (*χ*^2 ^= 50.429, *P* < .001).

**Table 1:** Sociodemographic characteristics of recruited participants.

	Municipality *N* = 281	Gyms *N* = 45	Social media *N* = 71	Family + friends *N* = 10	*P*	Total *N* = 407
Gender	_ _	_ _	_ _	_ _	<.001*	
Male, *N* (%)	142 (50.5)^a^	15 (33.3)^a^	8 (11.3)^b^	3 (30.0)^a,b^		168 (41.3)
Female, *N* (%)	139 (49.5)^a^	29 (64.4)^a^	63 (88.7)^b^	6 (60.0)^a,b^		237 (58.2)
Other, *N* (%)	0 (0.0)^a^	1 (2.2)^a,b^	0 (0.0)^a^	1 (10.0)^b^		2 (0.5)
Age in years, mean (SD)	64.0 (8.6)^a^	61.7 (8.5)^a,b^	60.0 (7.0)^a^	61.3 (9.5)^a,b^	.003*	63.0 (8.4)
Educational level	_ _	_ _	_ _	_ _	<.001*	
Low, *N* (%)	128 (45.6)^a^	8 (17.8)^b,c^	9 (12.7)^c^	5 (50.0)^a,b^		150 (36.9)
Middle, *N* (%)	103 (36.7)^a^	15 (33.3)^a^	29 (40.8)^a^	2 (20.0)^a^		149 (36.6)
High, *N* (%)	50 (17.8)^a^	22 (48.9)^b^	33 (46.5)^b^	3 (30.0)^a,b^		108 (26.5)
(Chronic) disease	_ _	_ _	_ _	_ _	.070	
Yes	154 (54.8)	18 (40.0)	29 (40.8)	4 (40.0)		200 (49.1)
No	127 (45.2)	27 (60.0)	42 (59.2)	6 (60.0)		207 (50.9)

^a,b,c^ Each superscript letter denotes a subset of group categories whose column proportions do not differ significantly from each other at the .05 level.

* *P* ≤ .05.

### 3.3 Which recruitment strategy is most suitable for reaching populations with a specific gender, age or health status?

Table 1 shows that with regard to gender, an almost equal amount of males (*N* = 142, 50.5% of the municipality group) and females (*N* = 139, 49.5%) was reached via the municipality. Significantly more males were reached via the municipality compared to gyms (*N* = 15, 33.3%) and social media (*N* = 8, 11.3%) (*χ*^2 ^= 60.546, *P* < .001). Further, participants reached via social media were significantly younger than participants reached via the municipality (mean age of 60.0 vs. 64.0 years) (F = 4.812, *P* = .003). Proportionally more participants without a (chronic) disease were reached via gyms (60.0% of the gyms group), social media (59.2%) and family + friends (60.0%), whereas proportionally more participants with a (chronic) disease were reached via the municipality (54.8%). However, this difference did not reach significance (*χ*^2 ^= 7.056, *P* = .070).

### 3.4 Which recruitment strategy is most beneficial with regard to costs?

An overview of the number of participants reached per strategy in relation to recruitment costs is provided in Table 2. Costs for recruitment via the municipality were with a total of €2,142.37 ($2,341.06) and €7.62 ($8.33) per yielded participant the highest, which comprised the letter/envelope-, printing- and delivery costs for 3,417 personal invitation letters. With regard to social media, the first advertisement ran for 7 days, cost €48.95 ($53.74), reached 7,316 Facebook users and resulted in 167 clicks on the link leading to the registration website. The second advertisement ran for 5 days, cost €50.09 ($54.99), reached 5,624 Facebook users and resulted in 179 clicks on the link leading to the registration website. Total costs for the social media recruitment were thus €96.81 ($105.79) and €1.36 ($1.49) per yielded participant. The ratio between the number of potential participants reached with the strategy and the number that actually registered to participate was with 8.2% for the municipality higher compared to 0.5% for social media. No data were available for the number of potential participants reached via the other two recruitment strategies.

**Table 2:** Overview number of participants reached in relation to recruitment costs.

	Municipality	Gyms	Social media	Family + friends
Total recruitment costs			** **	
In Euros (€)	2,142.37	0	96.81	0
In Dollars ($)^a^	2,341.06	0	105.79	0
Potential participants reached, *N*	3,417	Unknown	12,940	Unknown
Total participants, *N*	281	45	71	10
Ratio reached/registered, %	8.2	Unknown	0.5	Unknown
Costs per participant				
In Euros (€)	7.62	0	1.36	0
In Dollars ($)	8.33	0	1.49	0
Low educated participants				
*N*	128	8	9	5
% of total	45.6	17.8	12.7	50.0
Costs per low educated participant				
In Euros (€)	16.73	0	10.76	0
In Dollars ($)	18.28	0	11.76	0

^a^ Values in Euros converted to Dollars based on exchange rate of 06/20/2023.

## 4 Discussion

### 4.1 Principal findings

**Figure 2:**
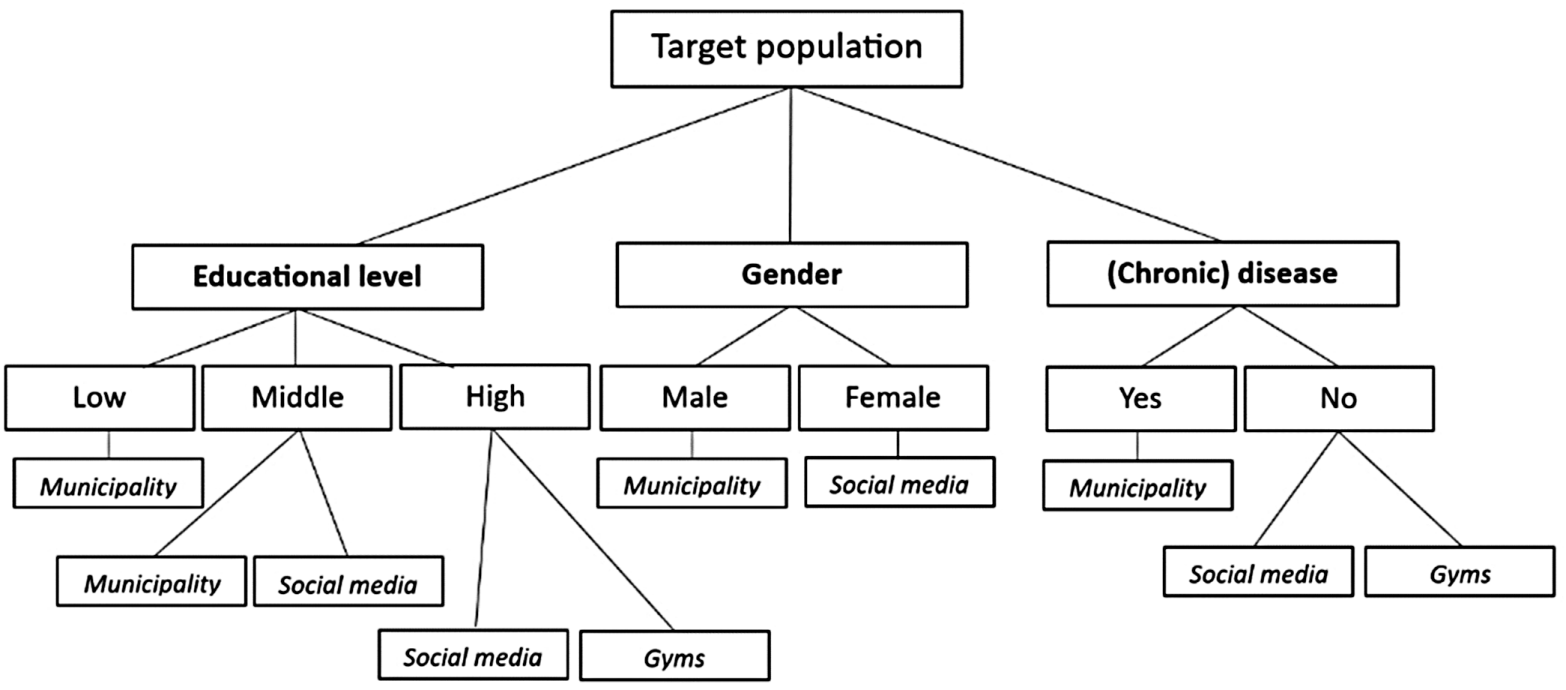
Decision tree for selecting a recruitment strategy to reach a specific target population of adults aged 50 years and older.

The aim of the current study was to provide insight into the reach, sample characteristics and costs of three pre-planned strategies for recruiting adults aged 50 years and older with low SES for participation in an online PA intervention including an activity tracker. The accompanying research questions can be answered based on the results of the study. Firstly, it can be concluded that recruitment via personal invitation letters through a municipality results in the highest and fastest response when compared to recruitment via gyms and social media. The observed highest reach/registered ratio for recruitment via the municipality is in line with other studies, showing that the application of more personalized approaches leads to higher enrollment rates (16) (RQ1). Additionally, the paper-based recruitment approach of sending personal invitation letters via the municipality was the most suitable for reaching the low SES population based on the strategies applied during the study (RQ2).. No paper-based recruitment approaches were applied for the gyms and social media since only online advertisements were used. Although the low SES population was thus quickly reached via the municipality, higher costs were involved for this paper-based strategy compared to the other completely online strategies. These higher costs need to be taken into account when selecting a recruitment strategy (RQ4). Figure 2 provides a decision tree for selecting the appropriate recruitment strategy in order to reach populations with specific sociodemographics based on the relative results of the study (RQ3). In particular, the result that relatively a larger amount of males (50.5%) was reached via the municipality approach is relevant since previous eHealth-studies have shown that males are more difficult to reach for participation in lifestyle-related interventions compared to females (17). The same applies to the findings on educational level, since low-educated participants are more difficult to recruit than middle- and high-educated participants (18). The decision tree guides future eHealth-studies in selecting an appropriate recruitment strategy.

### 4.2 Strengths

The application of different recruitment strategies parallel to each other can be considered a strength of the study. With this method, the target sample size of 400 participants aged 50 years and older was achieved within 10 weeks. This is a short period of time when compared to other eHealth-studies, where problems with reaching the sample size are frequently reported (19). The fact that participants received an intervention consisting of three tailored online PA advices combined with an activity tracker, which they were allowed to keep after completion of the study, contributed possibly to quickly reaching the desired sample size. However, considerations for participants to register were not investigated. More insight into reasons to participate could be valuable.

Further, the selected recruitment strategies can be considered successful since the population with low educational level was reached within this study. In particular, relatively large amounts of low educated participants were recruited via the municipality approach. Since the selected municipality has one of the lowest SES scores within the Netherlands (20), it is expected that also the low SES population was reached during recruitment. The additional procedures that were followed within the different recruitment strategies likely contributed to successfully reaching the low SES population as well. Among others, recruitment was deployed in low SES regions and targeting variables were added to social media advertisements. These additional actions are considered essential for reaching the low SES population. The sample characteristics of our preceding RCT underline this need, since mainly high-educated participants were reached while applying a more general recruitment strategy [submitted as Collombon EHGM, Bolman CAW, de Bruijn GJ, Peels DA, Verboon P, Lechner L: The efficacy of online physical activity interventions with added mobile elements within adults aged 50 years and over: a randomized controlled trial.], which is in line with other studies (13).

Lastly, the practical study design of this field study and the accompanying high external validity can be considered a strength. Commonly, recruitment studies present results of experimental study designs (7) which impedes the application of these results in practice.

### 4.3 Limitations

During this study, only educational level was assessed as outcome measure of SES. A more extensive insight into SES would have been obtained by assessing other factors as well (1, 21). An example is financial status, although it is expected that questions related to financial situation are not accepted by participants, as shown in previous studies (22). Since recruitment was employed specifically in low SES regions (municipality and gyms approach) it is expected that the low SES population was also reached while reaching low educated participants. However, this assumption should be interpreted with caution.

Further, it should be taken into account that this study was conducted in the Netherlands. The findings on recruitment strategies are only generalizable to other countries with caution. Comparable follow-up studies in other countries are recommended to confirm or refute our findings.

Although this study provides guidelines for reaching the low SES population, successful recruitment strategies alone are not sufficient. After registration, it is important that participants actually use an eHealth intervention. To encourage this, it is important that the characteristics and needs of the low SES population are taken into account during the design process of an intervention (23). It has namely been shown that this population faces more frequently low eHealth-literacy which comprises “a set of skills and knowledge that are essential for productive interactions with technology-based tools’’ (24–26). Populations with low eHealth-literacy are often not involved in research (27). Additionally, the low SES group has different life situations and eHealth expectations compared to the high SES group (28). Neglecting these differences during eHealth design processes can even exacerbate the digital divide and health inequalities instead of bridging this gap (29).Involving the target population in intervention design processes presents a solution for this (30).

### 4.4 Future recommendations

Based on the results of this study, future eHealth-studies are recommended to recruit via personal invitation letters through a municipality in order to reach the low SES population. It remains unclear whether solely the sending of paper-based personal invitation letters, solely approaching the target population via a municipality or the combination of both was responsible for successfully reaching the low SES group via this recruitment strategy. Future research could elucidate this. The degree of personalization could be further optimized by having contact with potential participants via telephone or face-to-face instead of sending personal invitation letters. Future studies are recommended to investigate whether more personalized approaches yield more low SES participants. However, it has to be taken into account that this is more time-consuming and costly compared to solely sending personal invitation letters.

Recruitment via family + friends was not a pre-planned strategy during this study and arose naturally. Future studies are recommended to investigate whether a pre-planned strategy via family and friends through snowball sampling has potential for reaching low SES participants. Although snowball sampling is commonly used within qualitative research (31), it might also be useful for reaching potential participants for participation in an online PA intervention.

### 4.5 Conclusions

In conclusion, recruitment via personal invitation letters through a municipality has the highest potential for reaching low SES participants of the three strategies applied during this study. However, higher costs are involved compared to recruitment via gyms and social media. Revealed insights on the sociodemographics gender, educational level and health status per recruitment strategy can guide future eHealth-studies to select appropriate strategies for reaching their specific target population of adults aged 50 years and older.

## Data Availability

The raw data supporting the conclusions of this article will be made available by the authors, without undue reservation.
